# Synthesis and Self‐Assembly of Pore‐Forming Three‐Arm Amphiphilic Block Copolymers

**DOI:** 10.1002/marc.202500077

**Published:** 2025-02-24

**Authors:** Sebastian Pusse, Bart‐Jan Niebuur, Tobias Kraus, Volker Presser, Bizan N. Balzer, Markus Gallei

**Affiliations:** ^1^ Department of Chemistry Saarland University 66123 Saarbrücken Germany; ^2^ Saarene Saarland Center for Energy Materials and Sustainability Campus C4 2 66123 Saarbrücken Germany; ^3^ INM–Leibniz‐Institute for New Materials Campus D2 2 66123 Saarbrücken Germany; ^4^ Colloid and Interface Chemistry Saarland University Campus D2 2 66123 Saarbrücken Germany; ^5^ INM–Leibniz Institute for New Materials 66123 Saarbrücken Germany; ^6^ Department of Materials Science & Engineering Saarland University 66123 Saarbrücken Germany; ^7^ Institute of Physical Chemistry University of Freiburg Albertstr. 21 79104 Freiburg Germany; ^8^ Cluster of Excellence livMatS @ FIT‐Freiburg Center for Interactive Materials and Bioinspired Technologies University of Freiburg Georges‐Köhler‐Allee 105 79110 Freiburg Germany; ^9^ Freiburg Materials Research Center (FMF) University of Freiburg Stefan‐Meier‐Str. 21 79104 Freiburg Germany

**Keywords:** amphiphilic polymers, block copolymers, pore formation, self‐assembly, star‐shaped polymers

## Abstract

The synthesis of an amphiphilic three‐arm block copolymer (AB)_3_‐BCP, which consists of poly(methyl methacrylate) (PMMA) and poly(butyl methacrylate) (PBMA) in the hydrophobic inner block, is reported. The hydrophilic block segment is based on poly(2‐hydroxyethyl methacrylate) (PHEMA) originating from 2‐(trimethylsiloxyl)ethyl methacrylate (HEMA‐TMS). The preparation is carried out in two steps using a core‐first approach. Using atom transfer radical polymerization (ATRP) as a controlled polymerization technique, three (AB)_3_‐BPCs with HEMA contents of 15 to 38 mol^−1^ % are prepared, applying different reaction conditions. Porous structures are generated from these BCPs by applying a self‐assembly and nonsolvent‐induced phase separation (SNIPS) protocol. Complex surface structures are observed using scanning electron microscopy (SEM). Bulk morphologies are investigated for a better understanding of the underlying self‐assembly. For PHEMA‐rich (AB)_3_‐BCPs, non‐regular lamellar microphases are observed in transmission electron microscopy (TEM) and confirmed by small‐angle X‐ray scattering (SAXS). The porous structures and their expected swelling characteristics are analyzed using atomic force microscopy (AFM) in air and water. Time‐resolved measurements in water indicate a rapid swelling after immersion into the water bath. The present study paves the way for exciting porous materials based on the herein synthesized amphiphilic three‐arm block copolymers useful for applications as absorber materials and coatings.

## Graphical abstract

A novel two‐step synthesis of an amphiphilic three‐arm block copolymer and self‐assembly and its tendency to pore formation is presented. Self‐assembly in the bulk state is investigated using TEM and SAXS. Pore formation following a SNIPS protocol is studied using SEM. The porous structures are further investigated via AFM in air and water.

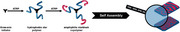

## 1 Introduction

Self‐assembly of block copolymers (BCPs) and their capability of microphase separation is one of the most intriguing features in polymer science. By connecting two immiscible polymers via a covalent bond, both block segments still follow their intrinsic urge to de‐mix into separated phases, as it is well known for blends of two or more immiscible polymers. The self‐assembly of BCPs and the resulting morphology in the bulk state at nanometer length scales are related to different properties of the underlying block copolymer, such as the degree of polymerization *N*, the enthalpic interaction between the different blocks, described by the flory‐huggins interaction parameter χ, as well as the volume fraction *f*.^[^ ^1^ ^]^ By varying these parameters different morphologies can be achieved. The dependence of the obtained morphology on the determining parameters has first been shown by leibler in a Mean‐Field‐Diagram.^[^ ^2^ ^]^ By increasing the volume fraction of B (*f*_B_), the obtained structures range from sphere‐like, cylindrical, and gyroidal to lamellar morphologies. The symmetrical phase diagram of BCPs can be distorted by tuning the molecular architecture of the BCPs. The tremendous influence of the underlying BCP architecture on self‐assembly capabilities was first investigated in the 80s and 90s of the last century. By synthesizing star‐shaped BCPs, where each arm can be interpreted as an AB‐block copolymer, the authors were able to shift and influence the bulk morphology from lamellae toward a bicontinuous structure by varying the degree of polymerization *N* as well as the number of arms.^[^ ^3^, ^4^, ^5^ ^]^ The higher complexity of the BCP architecture is transferred to the interphase between phases A and B in the bulk state by a change in its morphology. To minimize the interface interaction between both phases, the interphase favors a more curved shape.^[^ ^6^ ^]^ This leads to an asymmetric Mean‐Field‐Diagram. Distinct morphologies can now be stabilized over a wider range in the phase diagram of BCPs, which otherwise would not exist for linear BCPs. One example is the gyroidal morphology, which is typically only obtained in a narrow range of block composition for linear block copolymers; by varying the BCP architecture, this phase can be more accessible, for example, based on star‐shaped BCPs. Recently, we have developed different dendrimer‐like BCPs using different motifs as a central architecture and investigated their self‐assembly behavior as well as their influence on polymer blend systems.^[^ ^7^, ^8^ ^]^ These delicate architectures are based on convenient monomers like styrene and butadiene,^[^ ^7^ ^]^ or styrene and methyl methacrylate, where also a three‐arm motif as a central architecture is used.^[^ ^8^ ^]^ The convenient molecular variations enabled access to complex morphologies, which have formed the basis for different mechanical^[^ ^9^, ^10^ ^]^ as well as optical^[^ ^11^ ^]^ properties in materials.

By enlarging the pallet of monomers with hydrophilic properties, interesting materials can be prepared based on amphiphilic block copolymers. A prominent example of a hydrophilic monomer is 2‐hydroxyethyl methacrylate (HEMA).^[^ ^12^, ^13^, ^14^, ^15^ ^]^ A relevant application for this monomer acting as the hydrophilic part in amphiphilic BCPs is found in the field of ultrafiltration (UF) membranes.^[^ ^14^, ^15^ ^]^ Typically, the hydrophobic block segment of the BCP serves as a matrix and the hydrophilic minor block (e.g., HEMA) acts as a pore‐building block providing accessible hydroxy groups at the interior of the pores. Such functional groups can pave the way toward porous materials with highly sophisticated and feasible functionalities via postmodification.^[^ ^16^, ^17^, ^18^ ^]^ The second and most important aspect for applications in filtration systems, e.g., for wastewater treatment, are anti‐fouling properties accomplished by the implementation of hydroxy‐moieties on the surface of the membrane.^[^ ^19^, ^20^ ^]^ A feasible method for the preparation of BCP‐based UF membranes is the so‐called self‐assembly and non‐solvent induced phase separation (SNIPS) process,^[^ ^21^ ^]^ where the hydrophilic block collapses and unveils a monodisperse porous structure with a distinct cut‐off. Beneficial for this method is the precise adjustment of the cut‐off due to the linear correlation between chain length and pore size.^[^ ^22^ ^]^ Such porous materials are usually based on self‐assembly in a hexagonal cylindrical morphology.^[^ ^21^ ^]^ A prominent example is the preparation of a highly ordered UF membrane in a SNIPS process using a linear amphiphilic diblock copolymer of polystyrene and poly(2‐hydroxyethyl methacrylate).^[^ ^23^ ^]^ A drawback of previously reported linear block copolymer systems is the orientation of these cylindrical pore domains. While a perpendicular orientation concerning the material surface is mandatory in membrane applications, a horizontal alignment is thermodynamically preferred.^[^ ^24^ ^]^ To overcome this flaw of the linear BCP‐based approach, where the porous structure needs to be trapped kinetically to ensure a suitable top layer of the membrane, interconnected gyroidal porous structures could be feasible.^[^ ^24^, ^25^ ^]^ Such anisotropic porous structures are more difficult to obtain using linear BCPs because this morphology is only metastable in a certain range of phase‐determining parameters (degree of polymerization *N*, volume fraction *f*, interaction parameter *χ*). A broader range for the phase‐determining parameters and therefore a more accessible gyroidal morphology could be achieved with the use of an amphiphilic three‐arm BCP. The usage of this architecture could also achieve a higher cost‐efficiency in industrial‐scale membrane preparation because cheaper polymerization techniques like controlled radical polymerizations could be used instead of more cost‐intensive anionic polymerization strategies.^[^ ^26^ ^]^ NIPS‐based membranes, another suitable approach toward interconnected porous structures,^[^ ^27^, ^28^ ^]^ could be another cost‐efficient approach, but a monodisperse pore size distribution would not be maintained in this approach compared to the SNIPS‐based proposal using star‐shaped BCPs. To further improve UF membrane performance, interconnected porous structures can increase other parameters, such as the flux of such systems, compared to hexagonal cylindrical templated membrane systems.^[^ ^25^, ^29^ ^]^ In terms of long‐term stability, this approach could also be beneficial to the linear BCP system. Since the cylindrical pores with perpendicular orientation are kinetically trapped, the system may switch to a thermodynamically favorable orientation. The perpendicular‐oriented pores could start to tilt over time.^[^ ^30^ ^]^

## 2 Results and Discussion

**Scheme 1:**
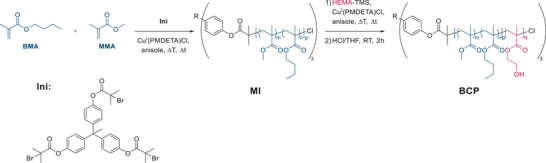
Synthetic route for the preparation of A3‐P(BMA‐co‐MMA)‐b‐P(HEMA) in a two‐step ATRP. First, a three‐armed star polymer containing BMA and MMA was prepared, to lead to the macroinitiators MI1 and MI2. In the second step, this star‐shaped copolymer is used as a macroinitiator for ATRP of HEMA‐TMS to generate a star‐shaped block copolymer.

**Figure 1:**
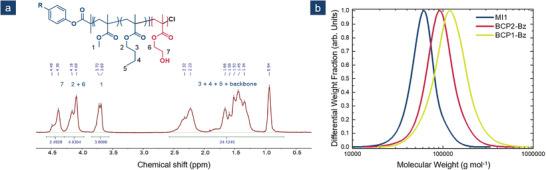
^1^H‐NMR spectrum of BCP3 in CDCl_3_ a) and SEC measurements of MI2 (blue) as well as BCP2 (red) and BCP3 (yellow) after Bz‐ protection in THF as an eluent b). [Correction added on February 26, 2025, after first online publication: Figure 1 has been revised].

In this work, we present a core‐first approach for the preparation of an amphiphilic (AB)_3_‐star‐shaped block copolymer using atom transfer radical polymerization in two stages. For the inner hydrophobic block, butyl methacrylate (BMA) and methyl methacrylate (MMA) were copolymerized statistically. The outer block was prepared from 2‐(trimethylsiloxyl)ethyl methacrylate (HEMA‐TMS) with an additional deprotection after synthesis, revealing a more hydrophilic segment as a second block. The amphiphilic star‐shaped block copolymer was investigated in its self‐assembly behavior as well as in a potential template for porous systems with complex structures using the hydrophobic block as a matrix building block and PHEMA as a pore‐forming block segment. The amphiphilic (AB)_3_‐BCP (poly (butyl methacrylate‐*co*‐methyl methacrylate)‐*b*‐poly(2‐hydroxyethyl methacrylate)) (AB)_3_‐P(BMA‐*co*‐MMA)‐*b*‐P(HEMA) with different fractions of the hydrophilic P(HEMA)‐block was prepared using atom transfer radical polymerization (ATRP), following a two‐step procedure, as shown in **Scheme** **1** and summarized in **Table** **1**. Starting from a tri‐functional initiator **INI**,^[^ ^31^ ^]^ a macroinitiator **MI** was prepared as a hydrophobic inner block, using ATRP for statistical copolymerization of BMA and MMA. The reaction was carried out using anisole as a solvent. For the catalyst, CuCl was chosen with *N,N,N’,N’’,N’’*‐pentamethyl diethylenetriamine (PMDETA) as a ligand. Two different macroinitiators, **MI1** and **MI2,** were prepared, varying the reaction temperature and reaction time. In both entries, polymers with monomodal distributions were received, showing a low dispersity of *Đ* = 1.13 with 56100 g mol^−1^ for **MI1** (**Figure** **1**a; Table S1, Supporting Information) and *Đ* =  1.13 with 50700 g mol^−1^ for **MI2** (Figure S2 and Table S1, Supporting Information). All size exclusion chromatography (SEC) measurements were calibrated based on linear polystyrene standard. Therefore, all molecular weights are expected to be slightly under‐interpreted, as it is shown in several earlier studies in the field of star‐shaped polymers.^[^ ^32^, ^33^, ^34^ ^]^ Usually, in ATRP, a transition from CuBr in macroinitiator synthesis to CuCl for the preparation of a second block in BCP formation can be used to obtain more defined BCPs due to a slower propagation rate of chlorine.^[^ ^35^ ^]^ Here, the bromine was already replaced at the initiator site. The reason for that is that in earlier tests, the macroinitiators received from synthesis using CuBr as a catalyst have shown a bimodal distribution or strong shoulders in SEC measurements (Figure S3, Supporting Information). Given this observation, a halogen exchange was already carried out during the macroinitiator synthesis to ensure a uniform initiation of all three initiator sites. Both monomers BMA and MMA were used in a ratio of 1:1 in both BCP syntheses. The obtained ratio of the monomers in both macroinitiators **MI1** and **MI2** was determined by ^1^H‐NMR spectroscopy (Figures S4 and S5, Supporting Information). As an outcome, the ratio of BMA and MMA in the final macroinitiators was also reflected in the ratio of the monomer mixture, proving the desired monomer incorporation. MMA and BMA were chosen in a ratio of 1:1 to adjust the glass transition temperature, *T*_g_, of the matrix building block. Matrices with high glass transition temperatures provide more stable membranes, while membranes based on matrices with lower glass transition temperatures tend to increase fracture resistance.^[^ ^36^ ^]^ For both macroinitiators, glass transition temperatures of *T*_g,MI1_ = 72.1 °C and *T*_g,MI2_ = 68.5 °C were achieved (Figures S6 and S7, Supporting Information).

**Table 1:** Compiled data on the macroinitiators MI1 and MI2 as well as the block copolymers BCP1, BCP2, and BCP3.

Polymer	MI	*M*_n,BCP_[SEC]^a)^	*Đ* ^a)^	*M*_n,PHEMA_[NMR]	*X*[BMA]/*X*[MMA]/*X*[HEMA]^b)^
MI1	−	56 100	1.13	−	0.5/0.5/0
MI2	−	50 700	1.13	−	0.50/0.5/0
BCP1	MI1	115 400 (108 500)	1.68 (1.24)	37 000	0.32/0.30/0.38
BCP2	MI1	97 300 (85 300)	1.56 (1.18)	20 700	0.38/0.37/0.25
BCP3	MI2	65 900	2.34	9 700	0.43/0.42/0.15

^a)^ *M*_n_ for the BCPs given based on SEC using DMF as eluent. Molecular weights and dispersities from THF‐SEC are given in brackets;

^b)^ Molar fractions are obtained from NMR spectroscopy measurements.

Starting from macroinitiators **MI1** and **MI2**, the second block of the star‐shaped BCP was introduced again via ATRP. As shown in Scheme 1 and Table S2 (Supporting Information), the polymerization was carried out in the same setup as it was used for macroinitiator formation. CuCl was used in combination with PMDETA for the catalyst system, while anisole was used as the solvent. To implement amphiphilic properties, 2‐(trimethylsiloxyl)ethyl methacrylate (HEMA‐TMS) as a protected hydrophilic monomer was chosen. The reaction was carried out at different temperatures for 20 h. Surprisingly, there was no direct correlation between reaction temperature and the observed degree of polymerization observable. The entry of **BCP2**, prepared at 90 °C, resulted in a lower molecular weight as compared to **BCP3** (80 °C). This observation indicates a loss of control at elevated temperatures due to the increased probability of chain termination.

After deprotection of the raw product in an acidic environment, the target structures were obtained as amphiphilic (AB)_3_‐block copolymers (Scheme 1). A successful polymerization was confirmed with ^1^H‐NMR spectroscopy with the appearance of a characteristic signal at 4.39 ppm corresponding CH_2_‐unit next to the hydroxy moiety of the PHEMA side chain after polymer workup (Figure 1a; Figures S8–S10, Supporting Information). These observations could also be confirmed via SEC measurements, as shown in Figure 1b and Figure S13 (Supporting Information). Additionally, **BCP1** and **BCP2** were analyzed using DSC, receiving the appearance of an *T*_g,BCP1_ = 115.4 °C (Figure S12, Supporting Information) and *T*_g,BCP2_ = 114.9 °C (Figure S13, Supporting Information) corresponding to the PHEMA segment.

**BCP1** was received from **MI1** as an entry at a reaction temperature of 80 °C. Using NMR spectroscopy in combination with the molecular weight for **MI1** from SEC, a molecular weight of *M*_n,BCP1_(HEMA) = 37000 g mol^−1^ was determined. This represents a molar fraction of 38 mol^−1^ % based on NMR data. For better comparison of the prepared BCP with their corresponding macroinitiators, all polymers were characterized via SEC. All polymers were measured using dimethylformamide (DMF) as an eluent (Figure S12, Supporting Information). **MI1**, **BCP2**, and **BCP3** were additionally characterized via SEC using tetrahydrofuran (THF) as an eluent (Figure 1b). For this approach, the hydroxy moieties of **BCP1** and **BCP2** had to be protected using a method already introduced earlier by schöttner et al. for the modification of PHEMA in other block copolymers.^[^ ^23^ ^]^ As shown in Figure 1b, a significant shift between **MI1** (blue) and both block copolymers **BCP1** (yellow) and **BCP2** (red) were observed. For both BCPs, the SEC traces show a narrow and monomodal size distribution. As expected from NMR data **BCP1**, containing a larger amount of the second block, showed a stronger shift to higher molecular weights compared to **BCP2**, containing 25 mol^−1^ % HEMA over the whole molecular weight of the BCP.

Both BCPs show a second significantly smaller signal shift toward higher molar masses in SEC measurements using DMF (Figure S12, Supporting Information). These traces contribute to the total molecular weight distribution with 1.6% for **BCP2** and 4.7% for **BCP1**. These observations match our hypothesis of an increased termination rate at elevated temperatures for this system. For **BCP3,** only SEC measurements were taken using DMF as an eluent (Figure S12, Supporting Information). A relatively small shift from **MI2** to **BCP3** matches the trend given from **BCP1** and **BCP2**. The molecular weight distribution for **BCP3** shows a shoulder toward higher molecular weights. This could be connected to unfavorable side reactions at late‐stage propagation reactions. This observation matches the high dispersity of *Đ* = 2.34 in DMF. In general, the SEC measurements of the unprotected BCPs show broader distributions compared to the Bz‐protected block copolymers in THF. These observations can be connected to strong interactions of the hydroxy moieties. In our application, dispersity plays a decisive role as it can influence the self‐assembly of the BCP.^[^ ^30^ ^]^ It is to be mentioned that due to the interactions between hydroxy moieties in the second block, the dispersity will appear higher with a given SEC setup. Therefore, the values of the Bz‐protected samples are given in brackets, too. The latter values are more reliable as there is no interaction with the SEC columns, as shown in previous studies.^[^ ^14^, ^15^, ^23^ ^]^

**MI2** was used as a macroinitiator for **BCP2**, synthesized at 90 °C with a reaction time of 20 h. For **BCP2**, a molecular weight of *M*_n, BCP2_(HEMA) = 20700 g mol^−1^ was determined analogously to **BCP1**. This equals a molar fraction of *x*_BCP2_(HEMA) = 0.15. These results matched a comparably higher shift of **BCP2** compared to **BCP 1**. Here, a dispersity of *Đ* = 1.56 was obtained.

The volume fraction *f*, which is relevant for the self‐assembly behavior, is listed in Table S3 (Supporting Information). The volume fraction for each block copolymer was determined based on the densities for both block segments and the molecular weight for each block. For the hydrophilic PHEMA block, a density of 1.275 g cm^−3^ can be assumed.^[^ ^37^ ^]^ The density for the hydrophobic matrix building block was estimated by the densities of the respective homopolymers of PBMA^[^ ^38^ ^]^ (1.041 g cm^−3^) and PMMA^[^ ^39^ ^]^ (1.188 g cm^−3^). All calculations can be found in the (Equations S1 and S2, Supporting Information).

For the preparation of porous films based on **BCP1–BCP3** using the method of self‐assembly and non‐solvent induced phase separation process (SNIPS process), polymer solutions were prepared, using THF, DMF, and 1,4‐dioxane (DOX) in a ratio of THF/DMF/DOX:2/1/1.^[^ ^14^ ^]^ The composition of the polymer solution as well as all membrane preparation conditions are given in table (Table S3, Supporting Information). The polymer solutions for **BCP1–BCP3** were cast on a THF‐preconditioned polyester nonwoven using a doctor blade with a blade gap of 200 µm. After a short evaporation time, the BCP film was placed in a precipitation bath consisting of distilled water. After drying, the BCP films were characterized using scanning electron microscopy (SEM).

**Figure 2:**
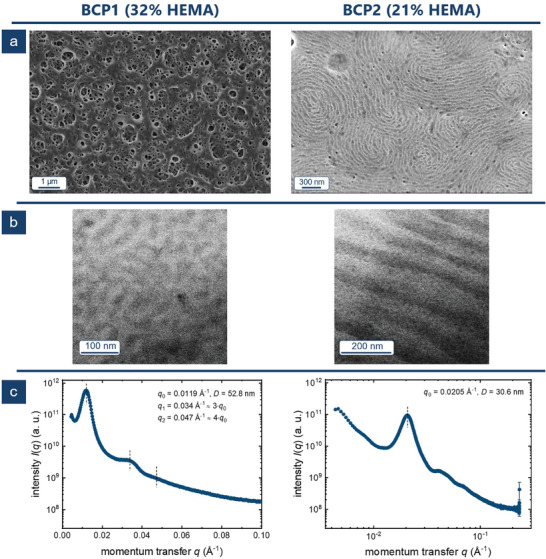
SEM images of the self‐assembly a), SEM images of porous structures based on BCP1 (left) and BCP2 (right) b), and SAXS measurements of the self‐assembly showing a main Bragg peak q_0_ and secondary Bragg peaks q_1_ and q_2_ c).

For **BCP3** with 14 vol% of PHEMA, only films with just a few pores were obtained. The pores appeared slightly tilted without any preferred orientation. This is in strong contrast to earlier studies with similar BCPs but with an underlying linear BCP architecture. In these former studies, the pores appeared more circular and had a perpendicular orientation referring to the material surface.^[^ ^14^, ^23^ ^]^ Additionally, a unique surface pattern was observed as well (Figure S14, Supporting Information). For the pore size, a pore diameter of *d*_BCP3, SEM_ = 40.2 ± 10.2 nm was determined, using 63 measuring points. To summarize the average pore diameter different pores were picked and measured at random angles using SEM micrographs. Compared to these findings, membranes based on **BCP2** with 25 vol% of PHEMA did not show any pores at all. (**Figure** **2**a, right) However, the SEM micrographs show a similar pronounced surface structure, indicating a strong tendency for self‐assembly in a structure by having no favored orientation at all. By increasing the amount of pore building PHEMA‐block to 32 vol%, a highly porous surface structure was received based on **BCP1**. (Figure 2a, left) As presented for **BCP3**, the pore diameter was determined based on 100 pores for an average of *d*_BCP1,SEM_ = 45.2 ± 10.6 nm. This observation meets the findings presented earlier where the pore size correlates with the length of the pore building block in the BCP.^[^ ^22^ ^]^

To gain more insights into these first findings from SEM, the polymers were further investigated in their bulk state. For this purpose, thin films of the bulk material were prepared. The thin films of the three polymers were analyzed using transmission electron microscopy (TEM). Dichlorodimethylsilane (Me_2_SiCl_2_) was used to stain the PHEMA block selectively to enhance the contrast for the assumed microphases for TEM. **BCP3** did not show any self‐assembly in this approach. **BCP3** did not self‐assemble into a classically ordered morphology in this approach, as shown by SAXS measurements. As shown in the corresponding scattering pattern of BCP3 (Figure S16, Supporting Information), a primary Bragg peak located at *q*_0_ = 0.0262 Å^−1^ indicates the presence of a microphase‐separated structure with a repeat distance of ≈24 nm. The absence of pronounced secondary Bragg peaks shows that the domains present were mainly positioned randomly with respect to each other. However, the very weak and broad scattering signal in the scattering pattern at $\sqrt{3}q_{0}$ and $\sqrt{7}q_{0}$ suggest that some domains in the sample may be arranged in a weakly‐defined hexagonal lattice. Given these results, this approach was not pursued further. In contrast, TEM imaging revealed a lamellar morphology for **BCP2** (Figure 2b, right). The wavy lamellar pattern of the interface between the two phases is highlighted here. This could be an indication of the beginning of a phase transition from a lamellar morphology toward a bicontinuous morphology. The average repeat distance of the more complex morphology, based on TEM imaging, is 92.5 ± 17.1 nm. The distances measured are shown again in Figure S15 (Supporting Information) (left). It is to be mentioned here that this pattern was found only in certain areas, indicating only a weak long‐range order. For **BCP1** transmission electron micrographs, a lamellar pattern was revealed over the whole sample. An average repeat distance of 43.1 ± 3.8 nm was measured based on TEM. (Figure S15, Supporting Information, right) These results were confirmed by small‐angle X‐ray scattering (SAXS) experiments. (Figure 2c) For **BCP1**, a main Bragg peak *q*_0_ was received, corresponding to a domain size of 52.8 nm. The pattern of the secondary reflections *q*_1_ and *q*_2_ with *q*_1_ = 3*q*_0_ and *q*_2_ = 4*q*_0_ strongly suggests a lamellar structure. (Figure 2c, left) The domain size based on SAXS matches the domain size based on TEM quite well. By contrast, for **BCP2,** only a primary Bragg peak was observed in SAXS. (Figure 2c, right) Here *q*_0_ indicates a domain size of 30.6 nm as *D* = 2π/*q*_0_. Furthermore, no secondary Bragg peaks following a defined pattern, e.g., lamellar, gyroidal, or hexagonal cylindrical morphologies were observed. The on average considerably smaller domain size in SAXS and the unspecific SAXS pattern supports the observations from TEM, where only in small areas an irregular lamellar pattern was found. One explanation could be an indication of a mixed morphology in the sample.

**Figure 3:**
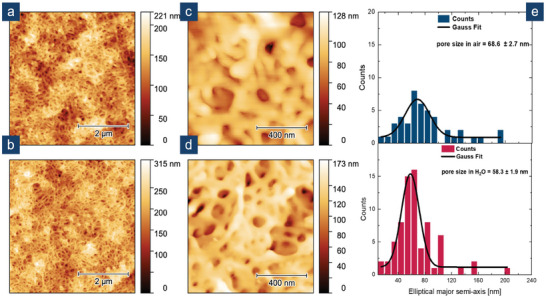
AFM‐based topography images of a BCP1‐membrane in air a,b) and water (ca. 167 min after immersion in water) c,d). Pore diameter distributions of b and d are given as Gaussian fits based on the elliptical major axis an average pore diameter e).

For further investigations on the pore formation process during the membrane‐building process and the pore development in aqueous media, **BCP1** membranes were examined in more detail by atomic force microscopy (AFM)‐ based imaging. **Figure** **3**a,b show the topography in air at a 5 µm scale and 1 µm scale. The topography of **BCP1** in water after an immersion time of almost 3 h is presented in Figure 3c,d, again at a 5 µm scale and 1 µm scale. In both cases, a sponge‐like structure is observed. Figure 3e represents in blue a pore diameter distribution based on the major axis of an elliptical fit for the sample in air. A similar distribution is presented in red for the sample in water. Average diameters for both distributions were determined by the application of a Gaussian fit, namely 68.6 ± 2.7 nm (air) and 58.3 ± 1.9 nm (water). In both cases, the pore diameter appears to be larger compared to the diameter determined using SEM. This could be explained by using the elliptical major axis as a measure for irregularly shaped pores. By contrast, for SEM, axes of random orientations were picked, possibly leading to a higher degree of averaging. Comparing the received pore diameters in air and water, only a small degree of swelling, with a loss of diameter on the order of 10% was observed using AFM. Due to a high affinity of PHEMA for water, a stronger degree of swelling had been expected. Furthermore, time‐dependent AFM‐based imaging was performed in water. There, over a time span of 27 to 161 min, the size of the pores did not change significantly (Figure S17, Supporting Information), revealing that any observed slight swelling might occur immediately after immersing the sample in water.

## 3 Conclusion

In conclusion, a synthetic protocol for the preparation of amphiphilic tri‐arm block copolymers (AB)_3_‐P(BMA‐*co*‐MMA)‐*b*‐PHEMA was introduced as a sequential core‐first approach using atom transfer radical polymerization as a controlled polymerization technique. With this protocol, star‐shaped BCPs with different HEMA contents could be synthesized. The influence of the implemented molecular architecture on the formation of porous structures was investigated following a SNIPS protocol. Here, different complex surface structures were observed. The underlying self‐assembly was further investigated by exploration of the morphology in bulk. To do so, thin polymer films were studied using TEM and SAXS revealing a lamellar morphology, comprising pronounced irregularities compared to typical lamellar structures. The respective porous material was further tested for potential applications in aqueous media. The estimated swelling property of the pore building HEMA‐block was analyzed using AFM in air and water as a medium. Here, a rapid swelling was observable. The approach presented here delivers membrane materials with complex porous structures. Those could be used as potential host systems for heterogeneous catalysis after postmodification of the pore‐building moiety. It could also be suitable as a blueprint for filtering applications, like ion harvesting or bio‐applications hosting enzymes.

## Conflict of Interest

The authors declare no conflict of interest.

## Supporting information



Supporting Information

## Data Availability

The data that support the findings of this study are available in the supplementary material of this article.
