# Supplementary material for: Synthesis and Self‐Assembly of Pore‐Forming Three‐Arm Amphiphilic Block Copolymers
Source: Macromol Rapid Commun. 2025 Feb 24;46(12):2500077. doi: 10.1002/marc.202500077 (PMC12183151; doi:10.1002/marc.202500077)
Supplement: Supplementary file 1 — Supporting Information [file MARC-46-2500077-s001.docx]

**Supporting Information**

Synthesis and Pore Evolution of Three-Arm Amphiphilic Block Copolymers

Sebastian Pusse, Bart-Jan Niebuur, Tobias Kraus, Volker Presser, Bizan N. Balzer*, and Markus Gallei*

Nuclear magnetic resonance (NMR)

NMR spectra were recorded on a Bruker Avance II 400 spectrometer with a 9.4 T Ultrashield Plus Magnet, a BBFO probe, and referenced by using the solvent signals.^1^ For processing and evaluation of the spectra MestReNova 14.2.0 was used.

Standard size exclusion chromatography (SEC)

SEC was performed with a 1260 Infinity II (Agilent Technologies) system and in this paper THF was used as the mobile phase (HPLC grade, flow rate 1 mL min^-1^) on an SDV column set from polymer standard service (PSS) (SDV 10^3^ A, SDV 10^5^A, SDV 10^6^ A, 5 µm) with a PSS SECurity^2^ RI/UV detector. Calibration was carried out using polystyrene (PS) standards from PSS. For Multi-Angle Laser-Light Scattering (SEC-MALLS) a PSS SLD 7000 detector was used. PSS WinGPC® UniChrom V 8.31 was used for data acquisition and evaluation of the measurements. With dimethylformamide (DMF) as the mobile phase (flow rate 1mL min^-1^, containing 1 g L^-1^ LiBr) a PSS GRAM Analytical column from PSS (10^3^ A) is used at a temperature of 60 °C. The Calibration for this set-up is based on poly(methyl methacrylate) (PMMA) standards from PSS.

*Scanning* electron microscopy (SEM)

SEM was carried out on a ZEISS GEMINI 500 microscopeusing the software SmartSEM Version 6.07. The samples were mounted on an aluminum stud using adhesive copper tape and sputter-coated with approximately 6 nm platinum using an Automatic Turbo Coater PLASMATOOL 125 SIN 2020_131 from Ingenieurbüro Peter Liebscher.

Differential scanning calorimetry (DSC)

DSC was carried out on a Netzsch DSC 214 Polyma with a heating rate of 20 K min^-1^ and nitrogen as both protective and purge gas in flow rates of 60 mL min^-1^ and 40 mL min^-1^, respectively. For evaluation NETZSCH Proteus Thermal Analysis 8.0.1 was used.

Atomic force microscopy (AFM)

For AFM-based imaging the Cypher ES (Asylum Research, an Oxford Instruments Company) was used in intermittent-contact mode (AC mode) with photothermal excitation at ca. 25 °C in air and ultrapure water (0.055 µS cm-1, Purelab Chorus 1, ELGA LabWater) with SCOUT 70 RAu cantilevers (NuNano, spring constant: ca. 2 N/m, resonance frequency in air: ca. 70 kHz, tip radius: ca. 5 nm). Further imaging parameters: 512∙512 pixels, scan rate: 2.44 Hz and scan angle: 90°, i.e., fast scan axis was perpendicular to the cantilever axis. The samples were glued on mounting pucks using double-sided carbon tape (Plano).

For evaluation, the images were processed via the Gwyddion Free SPM analysis software version 2.60^2^ applying the following operations in the presented order on the z sensor retrace data: shifting of minimum data values to zero (fix zero function). A linear color scale, gwyddion.net, was used for the presentation of the images. If necessary, the align rows (median difference), remove scars, and level (plane level and polynomial background) functions were applied. The pore diameters were obtained as described previously ^3^ In short: the Mask Editor tool was used to mask each pore manually. Then, each pore diameter was characterized by the elliptical major axis using the masked area. Histograms of the elliptical major axis were generated in IGOR Pro (Version 7, WaveMetrics), applying a Gaussian fit to obtain mean (maximum of fit) and error (half-width of the fit) values. Please note than any pore diameters below the cantilever tip radius of ca. 5 nm cannot be determined.

*Small-angle X-ray scattering (SAXS)*

SAXS experiments were performed using a Xeuss 2.0 lab-scale system (Xenocs SAS). A Copper K_α_ X-ray source (wavelength *λ* = 0.154 nm) was used as the incident beam, collimated and focused on the sample with a spot size of 0.25 mm^2^. 2D scattering intensity patterns were recorded using a Pilatus 300 K detector with pixel sizes of 172 × 172 μm^2^. The detector was located at a sample-detector distance of ~ 2500 mm, as calibrated using a silver behenate standard. All samples were measured for 3600 s. As no signs of anisotropic scattering were observed, the scattering patterns were azimuthally averaged to obtain the scattered intensity *I*(*q*), with *q* being the momentum transfer given by *q* = 4π sin(θ/2)/*λ*, where *θ* is the scattering angle.

The chemicals used were purchased from Sigma Aldrich, Acros Organics, Alfa Aesar and BLDpharm and used as received, unless otherwise stated. The monomers methyl methacrylate (MMA), butyl methacrylate (BMA) and 2-(trimethylsiloxyl)ethyl methacrylate (HEMA-TMS) were prepared before use by removing the stabilizer. For this purpose, the monomer was passed over an AlOx column. Cu^I^Cl was suspended in glacial acetic acid overnight, washed with ethanol (absolute) and dried in a high vacuum. The purified copper salt was stored in a glovebox until use.

*Synthesis of 1,1,1-Tris(4-(2-bromoisobutyryloxy)phenyl)ethane (****Ini****)^4, 5^*

First in a 500 mL three-necked round-bottom flask 2-bromoisobutyryl bromide (8.5 mL, 69.1 mmol, 4.1 eq.) was dissolved in dried THF (125 mL) and cooled using an ice bath. In the second step 1,1,1-tris(4-hydroxyphenyl)ethane (5.07 g, 16.7 mmol, 1 eq.) was dissolved together with triethylamine (13.7 mL, 6 eq.) in dry THF (110 mL) in a drip funnel. The solution was added dropwise to the cooled solution for over 45 minutes. After that, the solution was stirred for 2.5 days at room temperature.

The white solid that formed over the reaction was filtered off and the remaining solvent was removed under reduced pressure. The remaining brownish solid was dissolved in EtOAc and was extracted using aqueous NaOH (5%). The organic phase was again extracted using water. The solvent was removed under reduced pressure. The remaining brownish solid was recrystallized twice from EtOAc to receive a white powder (5.04 g, 55.7 %).

Figure S 1: NMR of **INI** in CDCl_3_.

^1^H-NMR (400 MHz, 300K, CDCl3): δ = 7.16 – 7.01 (m, 2H, 3H 18); 2.18 (s, 4H, 3); 2.07 (s, 1H, 18).

*Synthesis of A_3_-Poly(butyl methacrylate-co-methyl methacrylate)* *(A3-P(BMA-co-MMA))* **MI1** *and* **MI2**:

First, in a baked-out Schlenk flask, **1** was dissolved in anisole, followed by BMA as well as MMA. Then the solution was degassed by freeze-pump-thaw technique in three cycles. The reaction solution was heated up to 70°C afterward. The polymerization was initiated by adding 0.1 mol L^-1^ Cu^I^(PMDETA)Cl solution in anisole (1.00 mL). The reaction was terminated after several minutes. The polymer was precipitated from the reaction mixture in *n*‑hexane. Afterward, the polymer was dissolved in THF and passed twice through an aluminum oxide column (neutral). In the last step, the polymer was again precipitated in *n*‑hexane.

Table S 1: Synthetic approaches for MI1 and MI2.

|  | **MI1** | | | | **MI2** | | | |
| --- | --- | --- | --- | --- | --- | --- | --- | --- |
|  | *n*/mmol | *m*/g | *V*/mL | eq. | *n*/mmol | *m*/g | *V*/mL | eq. |
| Initiator | 0.0658 | 0.0496 | - | 1 | 0.0666 | 0.0502 | - | 1 |
| MMA | 65.4 | - | 7 | 994 | 67.8 | - | 7.25 | 1017 |
| BMA | 62.9 | - | 10 | 995 | 66 | - | 10.5 | 991 |
| Cu^I^(L)Cl^a,b^ | 0.195 | - | 1 | 2.96 | 0.2 | - | 1 | 3 |
| Cu^II^(L)Cl_2_^a,b^ | 0.0007 | - | 0.033 | 0.01 | - | - | - | - |
| Anisole | 147 | - | 16 | 2236 | 152 | - | 16.5 | 2278 |
| *T*/°C | 70 | | | | 55 | | | |
| *t*/min | 45 | | | | 165 | | | |
| *M*_n_/g mol^-1c^ | 56100 | | | | 50700 | | | |
| *Đ*^c^ | 1.13 | | | | 1.13 | | | |

*^a: L = PMDETA ;b: catalyst was prepared as a stock solution (c=0.2 M); c: SEC in THF.^*

Figure S 2: SEC curve of **MI2** in THF against PS calibration.

*Figure S 3: SEC curve of two exemplary entries based on CuBr in THF against PS calibration.*

Figure S 4: 400 MHz NMR spectrum of **MI1** in CDCl_3_.

^1^H-NMR (400 MHz, 300K, CDCl_3_): δ = 0.85 – 1.90 (backbone+3+4+5, m); 3.59 (2, s, 3H); 3.94 (1, s, 2H).

Figure S 5: 400MHz NMR spectrum of **MI2** in CDCl_3_.

^1^H-NMR (400 MHz, 300K, CDCl_3_): δ = 0.83 – 1.90 (backbone+3+4+5, m); 3.59 (2, s, 1H); 3.94 (1, s, 2H).

Figure S 6: DSC diagram of **MI1** between 40°C and 120°C at a rate of 10°C min^-1^ in nitrogen atmosphere.

Figure S 7: DSC diagram of **MI2** between 40°C and 120°C at a rate of 10°C min^-1^ in nitrogen atmosphere.

*Synthesis of A3-Poly(butyl methacrylate-co-methyl methacrylate)-b-P(2-hydroxyethyl methacrylate) (A3-P(BMA-co-MMA)-b-P(HEMA))* **BCP1 – BCP3**:

In a baked-out Schlenk flask, the macroinitiator was dissolved in anisole under an argon atmosphere. In the next step, (trimethylsilyloxyl ethyl) methacrylate was added. The mixture was degassed via freeze-pump-thaw technique in three cycles. The degassed solution was heated. The reaction was started with the addition of 0.1 mol L^-1^ Cu^I^(PMDETA)Br solution in anisole. The reaction was stopped by cooling down the flask and exposition of the reaction mixture to air. THF was added to the reaction mixture. The diluted mixture was passed through an aluminum oxide column (neutral). The HEMA-TMS block was deprotected by the addition of hydrochloric acid in THF (10 -30 mL, 2 mol L^-1^). The block copolymer was precipitated in *n*-hexane.

Table S 2: Synthetic approaches for BCP1, BCP2 and BCP3.

|  | **BCP3** | | | |
| --- | --- | --- | --- | --- |
| from | MI1 | | | |
|  | *n*/mmol | *m*/g | *V*/mL | eq. |
| MI | 0.0174 | 1 | - | 1 |
| HEMA-TMS | 11.5 | - | 2.5 | 659 |
| Cu^I^(L)Cl | 0.1 | - | 0.32 | 3.7 |
| Cu^II^(L)Cl_2_ | - | - | - | - |
| Anisole | - | - | 19.0 | - |
| *T*/°C | 55 | | | |
| *t*/h | 20 | | | |
| *M*_n_/g mol^-1^ | 9700 | | | |
| *Đ* | 2.34 | | | |
|  |  |  |  |  |
|  | **BCP2** | | | |
| from | MI1 | | | |
|  | *n*/mmol | *m*/g | *V*/mL | eq. |
| MI | 0.0174 | - | - | 1 |
| HEMA-TMS | 11.5 | - | 2.5 | 659 |
| Cu^I^(L)Cl | 0.1 | - | 0.32 | 3.7 |
| Cu^II^(L)Cl_2_ | - | - | - | - |
| Anisole | - | - | 19 | - |
| *T*/°C | 90 | | | |
| *t*/h | 20 | | | |
| *M*_n_/g mol^-1^ | 20700 | | | |
| *Đ* | 1.56 | | | |
|  |  |  |  |  |
|  | **BCP1** | | | |
| from | MI2 | | | |
|  | *n*/mmol | *m*/g | *V*/mL | eq. |
| MI | 0.0198 | 1 | - | 1 |
| HEMA-TMS | 11.9 | - | 2.6 | 603 |
| Cu^I^(L)Cl | 0.1 | - | 0.35 | 3.5 |
| Cu^II^(L)Cl_2_ | 0.0001 | - | 0.007 | 0.007 |
| Anisole | - | - | 21.5 | - |
| *T*/°C | 80 | | | |
| *t*/h | 20 | | | |
| *M*_n_/g mol^-1^ | 37000 | | | |
| *Đ* | 1.56 | | | |

Figure S 8: 400 MHz NMR spectrum of **BCP1** in pyridine-d_5_.

^1^H-NMR (400 MHz, 300K, Pyridine-d_5_): δ = 0.94 – 2.23 (backbone+3+4+5, m); 3.69 (3, s, 1H); 4.09 – 4.16 (2+6, m, 2H+2H); 4.39 – 4.49 (7, m, 2H); 5.04 (H_2_O).

Figure S 9: 400 MHz NMR spectrum of **BCP2** in pyridine-d_5_.

^1^H-NMR (400 MHz, 300K, Pyridine-d_5_): δ = 0.94 – 2.32 (backbone+3+4+5, m); 3.69 (3, s, 1H); 4.08 – 4.16 (2+6, m, 2H+2H); 4.38 – 4.49 (7, m, 2H); 5.01 (H_2_O).

Figure S 10: 400 MHz NMR spectrum of **BCP3** in pyridine-d_5_.

^1^H-NMR (400 MHz, 300K, Pyridine-d_5_): δ = 0.94 – 2.32 (backbone+3+4+5, m); 3.71 (3, s, 1H); 4.10 – 4.17 (2+6, m, 2H+2H); 4.39 – 4.49 (7, m, 2H); 5.56 (H_2_O).

Figure S 11: DSC diagram of **BCP1** between 0 °C and 200°C at a rate of 10°C min^-1^ in nitrogen atmosphere.

Figure S 12: DSC diagram of **BCP2** between 40°C and 120°C at a rate of 15°C min-1 in nitrogen atmosphere.

Figure S 13: Comparison of SEC measurements from **MI1**, **MI2**, **BCP1**, **BCP2** and **BCP3** using DMF as a solvent against a PMMA calibration.

*Calculation of the volume fraction of PHEMA:*

The volume for the hydrophobic block based on BMA and MMA is estimated by the following equations **eq. 1** and **eq. 2.** In both approaches represents *δ* the density and *m* the mass of the respective polymer.

|  | $\delta_{MI}=x_{BMA}\cdot\delta_{BMA}+x_{MMA}\cdot\delta_{MMA}$ | *eq. 1* |
| --- | --- | --- |
|  | $f_{HEMA}=\frac{\left( \frac{m_{HEMA}}{\delta_{HEMA}} \right)}{\left( \frac{m_{HEMA}}{\delta_{HEMA}} \right)+\left( \frac{m_{MI}}{\delta_{MI}} \right)}$ | *eq. 2* |

*Membrane Preparation*

The membranes shown in Figure 2c as well as Figure S 14 were prepared applying the following SNIPS preparation protocol:

For the solvent a stock solution was prepared containing THF, DMF and 1,4-dioxane (DOX) in a weight ratio of 2/1/1 (THF/DMF/DOX). The BCPs were dissolved over night while the polymer solution was continuously shaken. On this way a highly viscous and bubble free polymer solution was received. The solution was cast on a nonwoven made of polyester using a doctor blade (200µm). After a certain evaporation time the sample was carefully immersed into a precipitation bath for several minutes. Here water was used as a non-solvent. The membranes were dried under ambient conditions for several hours. For a last step, the membranes were further dried in a vacuum at 40°C overnight.

Table S 3: Compiled data for membrane preparation approaches of BCP1, BCP2 and BCP3.

| Block copolymer | *f*_HEMA_ | w-% of polymer in solution | Evap. time/  s | Temperature/  °C | Rel. humidity/  % |
| --- | --- | --- | --- | --- | --- |
| BCP1 | 0.32 | 28.1 | 15 | 22.6 | 35 |
| BCP2 | 0.21 | 28.4 | 20 | 22.3 | 34 |
| BCP3 | 0.14 | 25.0 | 20 | 22.7 | 33 |


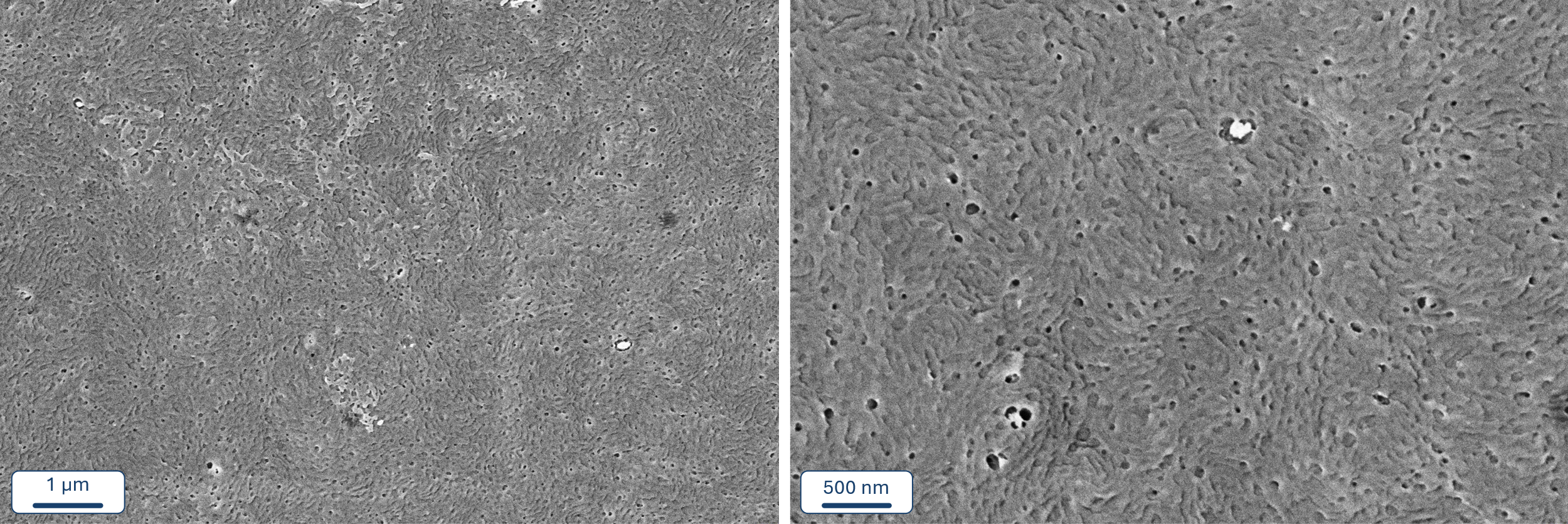


Figure S 14: SEM images of membranes based on **BCP3**.


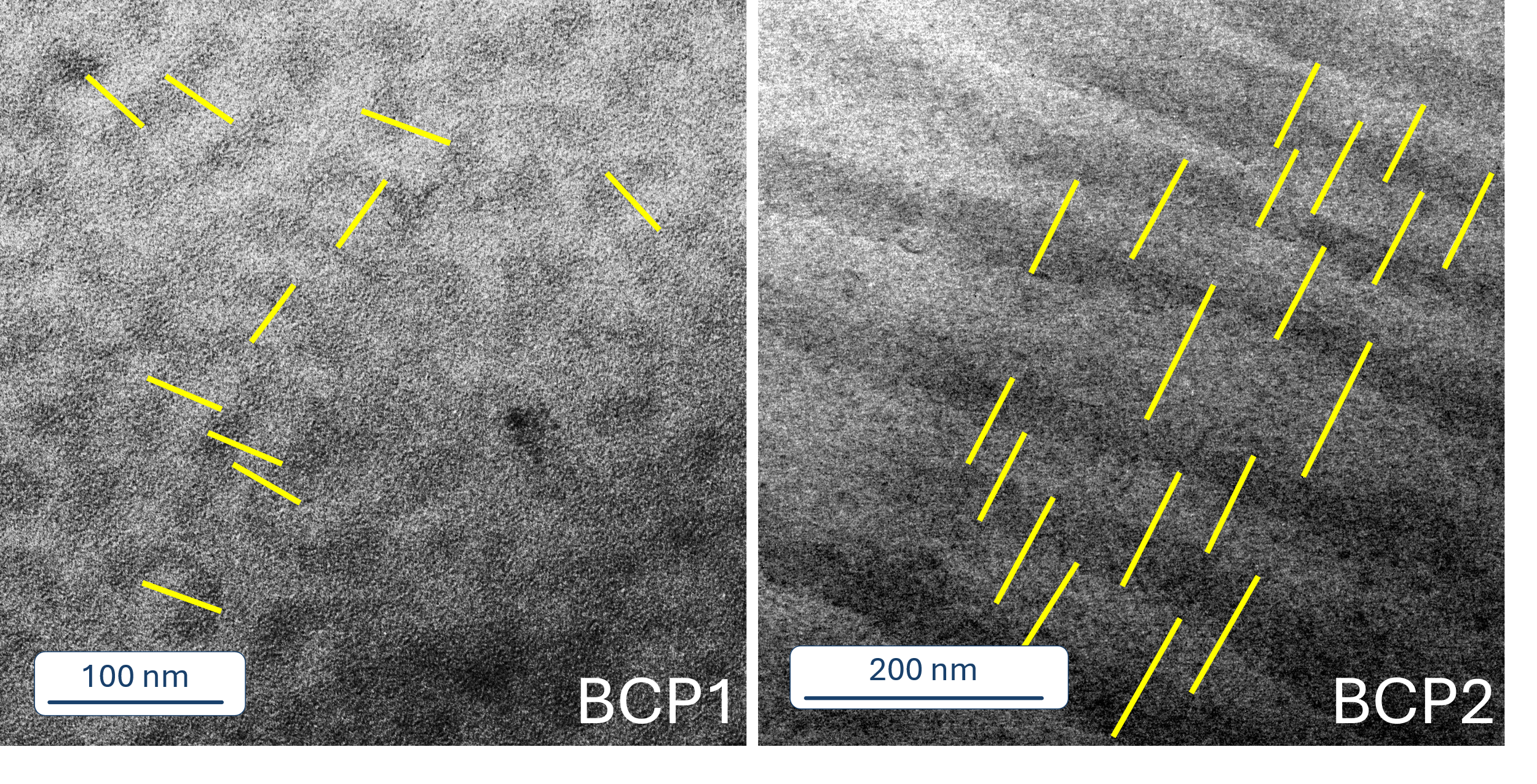


Figure S 15: TEM images of BCP1 and BCP2 with measurements highlighted for determination of the size of the repeating units.


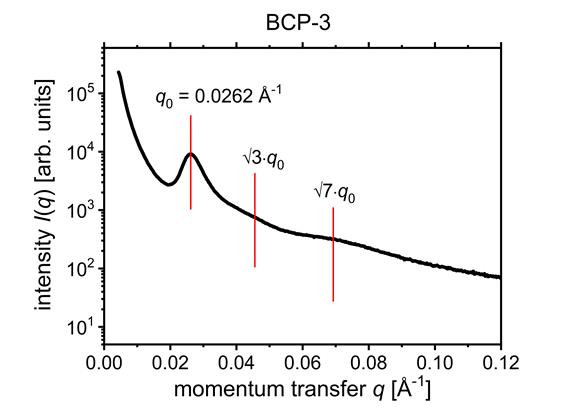


Figure S 16: SAXS measurements of the self-assembly showing a main Bragg peak q_0_ and secondary Bragg peaks q_1_ and q_2_.


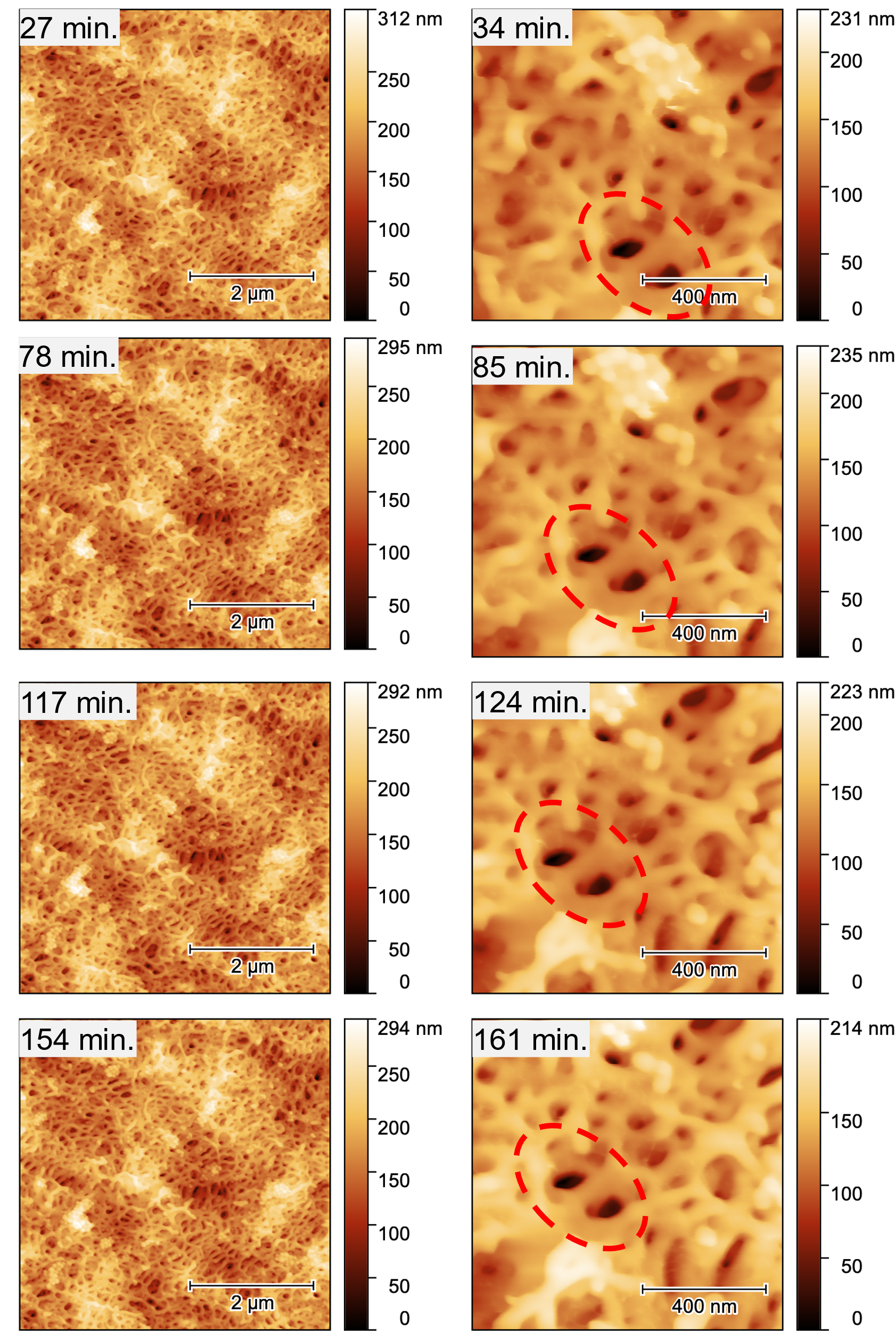


Figure S 17: AFM-based topography images of the BCP1-membrane over a time span of 27 to 161 minutes after immersion in water. Two exemplary pores are highlighted on the close-ups (1 x 1 µm^2^ scale, right side). The pores do not show any significant changes within the investigated timespan.

**References**

(1) Fulmer, G. R.; Miller, A. J. M.; Sherden, N. H.; Gottlieb, H. E.; Nudelman, A.; Stoltz, B. M.; Bercaw, J. E.; Goldberg, K. I., *Organometallics* **2010**, *29* (9), 2176-2179.

(2) Nečas, D.; Klapetek, P., *Open Physics* **2012**, *10* (1), 181-188.

(3) Friess, F. V.; Hu, Q.; Mayer, J.; Gemmer, L.; Presser, V.; Balzer, B. N.; Gallei, M., *Macromol Rapid Commun* **2022**, *43* (3), e2100632.

(4) Matyjaszewski, K.; Miller, P. J.; Pyun, J.; Kickelbick, G.; Diamanti, S., *Macromolecules* **1999**, *32* (20), 6526-6535.

(5) Porsch, C.; Zhang, Y.; Ducani, C.; Vilaplana, F.; Nordstierna, L.; Nystrom, A. M.; Malmstrom, E., *Biomacromolecules* **2014**, *15* (6), 2235-2245.
